# Association of soluble transferrin receptor/log ferritin index with all-cause and cause-specific mortality: National Health and Nutrition Examination Survey

**DOI:** 10.3389/fnut.2024.1275522

**Published:** 2024-02-27

**Authors:** Yan Yu, Dongying Lu, Zhenhui Zhang, Lili Tao

**Affiliations:** ^1^Guangzhou Baiyunshan Hospital, Guangzhou, China; ^2^Department of Pediatrics, Nanfang Hospital, Southern Medical University, Guangzhou, China; ^3^Department of Critical Care Medicine, The Second Affiliated Hospital, Guangzhou Medical University, Guangzhou, China

**Keywords:** soluble transferrin receptor, sTfR index, all-cause mortality, cardiovascular disease, cancer soluble transferrin receptor, cancer

## Abstract

**Background:**

Soluble transferrin receptor (sTfR)/log ferritin index (sTfR Index) can be used to assess the entire spectrum of iron status, and is valuable in evaluating iron status in population studies. There is still a lack of evidence on the association between sTfR index and all-cause mortality.

**Object:**

To explore the association between sTfR index and all-cause mortality, as well as mortality due to cardiovascular disease (CVD) and cancer.

**Method:**

Data were from the National Health and Nutrition Examination Survey (NHANES) between 2003 to 2020. Participants aged 16 years and older who had complete data of serum ferritin and sTfR were included. Pregnant individuals or those with ineligible data on death or follow-up were excluded from the analysis. Baseline sTfR index was calculated by baseline sTfR/log (ferritin) and classified as three tertile. We performed the Cox proportional hazard regression to assess the association of sTfR index (both continuous and categorical scale) with all-cause and cause-specific mortality and further assess the non-linear relationship between sTfR index and the outcomes with restricted cubic spline.

**Result:**

In this study, 11,525 participants, a total of 231 (2.0%) all-cause deaths occurred during a median follow-up of 51 months. The risk of all-cause mortality, CVD-related mortality, and cancer-related mortality was higher in participants with highest tertile of sTfR index. After confounding factors adjustment, participants with highest tertile of sTfR index were associated with an increased risk of all-cause mortality (HR: 1.71, 95% CI: 1.14–2.57) as compared with lowest tertile. Additionally, sTfR index per SD increment was associated with a 25% increasing risk of all-cause mortality (HR: 1.25, 95% CI: 1.08–1.45, *p* = 0.003) and a 38% cancer-related mortality (HR: 1.38, 95% CI: 1.07–1.77, *p* = 0.018). These associations remained robust after adjusting for the serum ferritin as well as in various subgroups stratified by age, sex, smoking statue, hypertension, diabetes, and CVD. Spline analysis showed that there is approximately linear relationship between sTfR index with all-cause mortality (*p* for non-linear = 0.481). Moreover, ferritin was not a predictor of all-cause death after adjustment for confounding factors.

**Significance:**

This cohort study demonstrated a significant association between sTfR index increment and an increased risk of all-cause and cancer-related mortality, independent of ferritin levels.

## 1 Introduction

The soluble transferrin receptor (sTfR) in serum is derived from the proteolysis of surface receptors on early erythrocytes, especially under conditions of low iron levels (1). It has significant application value in diagnosing iron deficient anemia (IDA) and serves as a sensitive and reliable indicator of functional iron deficiency (2). Ferritin is a cellular storage protein for iron, consisting of 24 subunits and its spherical cavity can store up to 4,500 iron atoms (3). However, the source of circulating ferritin and the pathway through which cells secrete ferritin are mostly unclear (4). Factors that affect serum ferritin expression include acute infection with concurrent inflammation (5), hemophilic lymphohistiocytosis (6) and cellular iron status, etc. Studies show that relying solely on ferritin level may delay diagnosis of combined IDA and anemia of chronic disease (7).

There are several ways to express the ratio of sTfR to serum ferritin (SF) as indicator of iron status (8): the logarithm of the ratio of sTfR to SF, the simple ratio of sTfR to SF, the ratio of sTfR to the logarithm of SF. The logarithm of the ratio of sTfR to SF concentrations, expressed as mg/kg body weight, known as the body iron index, is linearly related to total body iron stores (9, 10). It merely indicates the severity of the iron deficit at the low end of the spectrum and the magnitude of the iron surplus at the high end of the spectrum. The utility of the simple ratio of sTfR to SF concentrations (expressed in μg/L) is limited due to large differences in sTfR assays, and can only be used with data generated by an assay that performs equivalently to the Ramco or Roche assay (11, 12). Finally, the sTfR index, calculated as the ratio of sTfR to the logarithm of SF, was introduced as an indicator to identify persons with depleted iron stores (13, 14).

The sTfR index, which is superior to sTfR, improves detection of IDA, particularly in situations where routine markers provide equivocal results (7). sTfR index is calculated by dividing baseline sTfR by/log (ferritin). The sTfR index can be used to assess the entire spectrum of iron status, ranging from positive iron stores to negative iron balance, and is particularly valuable in evaluating iron status in population studies (15). The latest research indicates that sTfR index has greater diagnostic utility than sTfR in detecting iron deficiency anemia in the presence of chronic inflammation or discriminating iron deficiency without anemia (16, 17). sTfR index can also identify healthy subjects with subclinical iron deficits (18).

Maintaining iron homeostasis is essential for proper cardiac function and plays a crucial role in cancer and other diseases. An increasing body of research indicates that an iron imbalance is a common factor in many cardiovascular disease subtypes (1, 19). The elevated level of sTfR was linked to the prevalence of cardiovascular disease (20). It has also been shown that the expression of sTfR correlates with the occurrence of cancers (21) and tumor differentiation in breast, lung, and lymphoma cancers (22). Additionally, elevated sTfR levels are associated with an increased risk of developing T2DM in obese individuals (23), and represent an additional risk factor in systolic hypertension (24). Furthermore, sTfR index were significantly associated with 28 days mortality in sepsis patients admitted to the ICU (25).

However, the relationship between sTfR index and all-cause mortality, as well as mortality related to cardiovascular disease (CVD) and cancer patients, remains unclear. Therefore, we conducted this retrospective cohort study using data from the National Health and Nutrition Examination Survey 2003–2020 to investigate whether sTfR index can serve as an indicator for predicting the prognosis of diverse populations.

## 2 Methods

### 2.1 Study design and population

This is a prospective cohort study of a nationally representative sample of US adults using National Health and Nutrition Examination Survey (NHANES) from 2003–2010. NHANES is a comprehensive nationwide survey conducted by the National Center for Health Statistics (NCHS) at the Centers for Disease Control and Prevention (CDC). This survey involves home interview and collects data on various aspects including socioeconomic status, medical examinations, dietary habits, health information, as well as physical and physiological measurements of the U.S. population. NHANES is specifically designed to evaluate the health and nutritional status of both adults and children.

**Figure 1:**
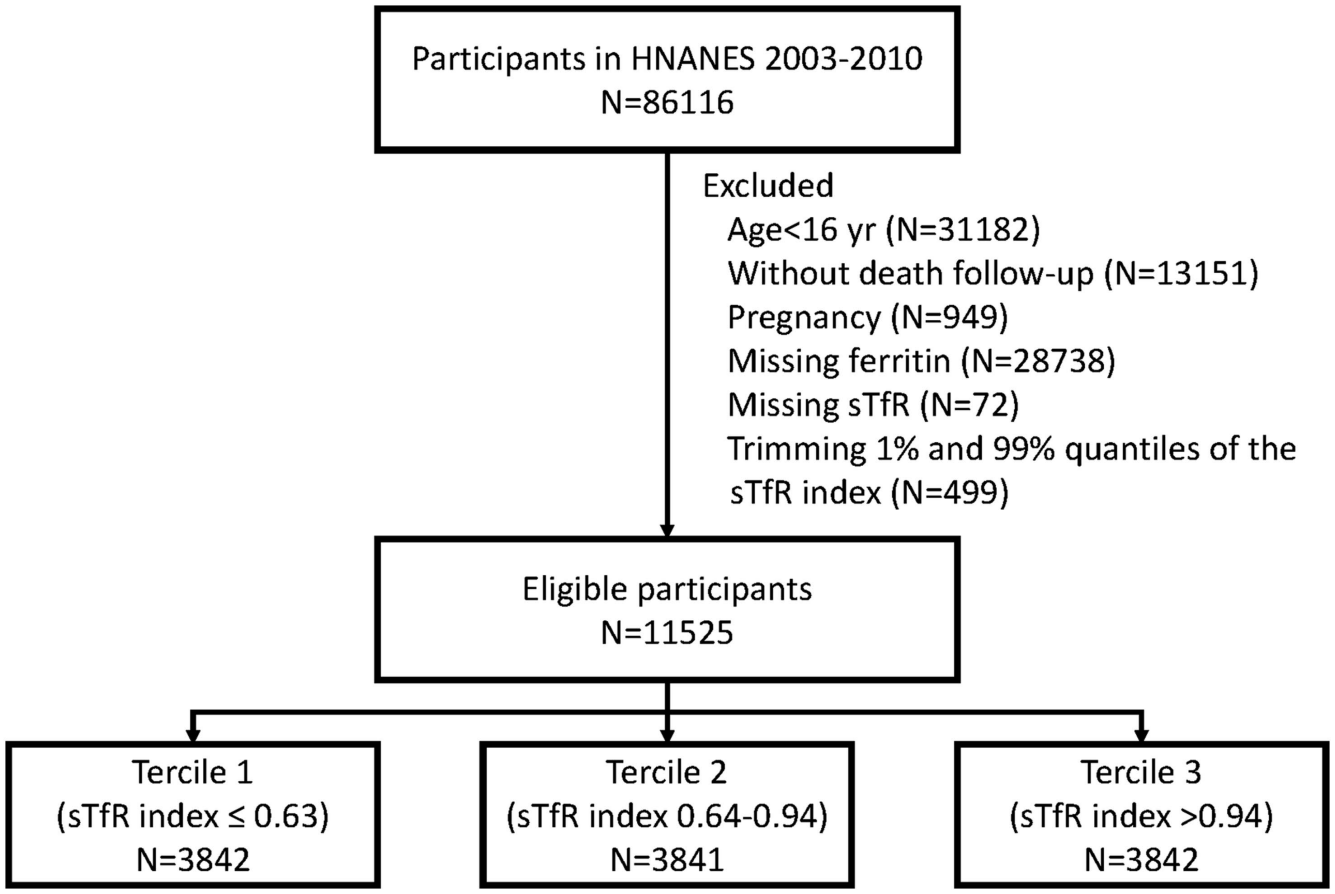
Study flowchart. sTFR, soluble transferrin receptor.

Figure 1 depicts the selection process of this study. Briefly, participants aged 16 years and older from the NHANES datasets spanning from 2003 to 2020 were included. We excluded participants with missing ferritin and sTFR data. Also, pregnant individuals and those who were not covered by the NDI were excluded from the analysis.

### 2.2 Exposure measurement

The primary exposure in this study was the sTfR index, calculated as baseline sTfR/log (ferritin) (3). sTfR index tertile assignment was as follows: tertile 1 (sTfR index ≤0.63), tertile 2 sTfR index (0.64–0.94), and tertile 3 (sTfR index >0.94). Ferritin was used as a measure of iron (Fe) stores, while serum soluble transferrin receptor (sTfR) was served as an indicator of Fe deficiency. The method measurement of Ferritin was performed using immuno-turbidimetry on the Roche/Hitachi 912 clinical analyzer. Latex-bound ferritin antibodies reacted with the antigen in the sample to form an antigen/antibody complex, and turbidimetric measurement was conducted after agglutination. The formed complexes were proportional to the ferritin concentration and were measured at a primary wavelength of 700 nm. The measurement of sTfR was conducted using a particle-enhanced immunoturbidimetric assay with Roche kits on the Cobas^®^ c501 clinical analyzer. Latex particles coated with anti-sTfR antibodies reacted with the antigen in the sample, leading to the formation of an antigen/antibody complex. After agglutination, the precipitate was photometrically determined.

### 2.3 Primary and secondary outcomes

Primary outcome was all-cause mortality; secondary outcomes included CVD-related mortality and cancer-related mortality. Mortality records, including death date and cause, were extracted from the National Death Index (NDI) for participants. Baseline data from NHANES 2003 to 2020 were linked to mortality data from the NDI death certificate records until December 31, 2019 to identify mortality status. Cause-specific deaths were determined using the International Statistical Classification of Diseases, 10th Revision (ICD-10). Cardiovascular death included rheumatic heart disease, hypertensive heart and renal disease, ischemic heart disease, heart failure, and cerebrovascular disease (054-064 in NCHS code). Cancer deaths included all malignant neoplasms (019-043 in NCHS code). The follow-up period was defined from the date of participants’ inclusion in the survey until the earliest of the last follow-up time (Dec 31, 2019) or the date of death. Participants not matched with a death record were considered alive through the entire follow-up period.

### 2.4 Covariates assessment

Baseline characteristics, including socioeconomic conditions (age, sex, ethnicity, and education), behavior, and history of diseases, were obtained through questionnaires. Body mass index (BMI) was calculated as weight divided by height squared (kg/m^2^) and categorized into five levels (<18.5, 18.5–24.9, 25.0–29.9, 30.0–34.9, and ≥35.0) (26). Drinking status was classified as ≥4 drinks per day, non-drinker or missing; smoking status was classified as current smoker, past smoker, non-smoker or missing. Laboratory results included serum albumin, total cholesterol, serum creatinine, serum potassium, hemoglobin, mean corpuscular volume (MCV), and ferritin. A history of hypertension or diabetes was defined based on self-reported physician diagnosis. The history of CVD was determined using self-reported questionnaires. Participants were asked five questions: “Has a doctor or other health professional ever told you that you have congestive heart failure, coronary heart disease, angina pectoris, heart attack, or stroke?.” Participants with the answer of “yes” to any question were considered to have CVD.

### 2.5 Statistical analyses

Baseline characteristics and laboratory measurements were presented as median (IQR) and frequency (%) for continuous and categorical variables. Group comparison was performed using Kruskal–Wallis and chi-square test, as appropriate. We conducted multivariable hazard proportional regression. Kaplan–Meier survival curves were plotted to calculate cumulative mortality using three tertile categories of sTfR index, and compared using the log-rank test. We utilized Cox proportional hazards regression models to assess the associations of sTfR index (both continuous and tertiles) with all-cause, CVD-related, and cancer-related mortality. To adjust for confounding factors, three model as fitted: Model 1 was adjusted for age (continuous), sex, and race/ethnicity; model 2 further adjusted for smoking status, drinking status, and education. In model 3 (primary analysis model), we additionally adjusted for BMI, SBP, DBP, albumin, total cholesterol, serum creatinine, serum potassium, Hemoglobin, MCV, diabetes, hypertension, CVD, and ferritin. The results of Cox regression were reported as hazard ratios (HRs) and their corresponding 95% confidence interval (CIs). Considering the potential non-linear relationship between sTfR index and all-cause mortality, we further performed a Cox regression with restricted cubic spline. We compared CVD-related and cancer-related mortality using Fine and Gray competing risks regression, with the creation of a cumulative incidence function. Non-cardiovascular and non-cancer mortality during the follow-up period were considered as competing risks, and patients lost to follow-up were censored. Sub-distribution hazard ratios (sHRs) and their corresponding 95% CIs were reported. Furthermore, we estimated the HRs of all-cause mortality, CVD-related mortality, and cancer-related mortality within 1, 5, and 10 years associated with sTfR index to examine the potential impact of sTfR index on short-term and long-term mortality. With respected to the cause-specific mortality in this study, we conducted an additional analysis to detect the associations between sTfR index (per SD increment) and mortality among patients with CVD and cancer.

Additionally, we classified patients into three groups based on the three tertiles of ferritin and examine the association between ferritin and all-cause mortality, CVD-related mortality, and cancer-related mortality using the same confounders adjustment mentioned above. For subgroups analyses, we assessed the association of sTfR index with all-cause mortality stratified by different effect modifiers, including age (≥40 and <40 years), sex, smoking status, hypertension, diabetes, CVD, and cancer. Interaction between TFR and pre-defined effect modifiers was fitted using the product term, and *p* < 0.05 indicated the significant effect modification. Considering the reverse causality, we conducted a sensitivity analysis to assess the sTfR index and study outcomes after excluding participants with baseline CVD or cancer.

Missing value were imputed by multiple imputation under the assumption of missing at random. Additionally, we conducted a sensitivity analysis after eliminating patients with missing values. A *p*-value less than 0.05 was considered statistically significant. All statistical analyses were performed using R software (version 4.1.1).

## 3 Results

### 3.1 Baseline characteristics of the study population

Overall, 28,738 and 13,151 participants were excluded due to the missing serum ferritin measurement and without death follow-up. Given to the potential selection bias, we further compared the characteristics among participants with and without serum ferritin measurement (Supplementary Table S1) and those with and without death follow-up (Supplementary Table S2). Finally, a total of 11,525 eligible participants were included in this study [median (IQR) age: 38 yr. (27–48); 80.4% were male] (Figure 1). The baseline characteristics of participants across three tertiles of sTfR index are presented in Table 1. The distribution of serum ferritin was showed in Supplementary Figure S1. Participants in tertile 3 (>0.94) of sTfR index were more likely to be younger, female, Non-Hispanic Black, and have a higher BMI.

**Table 1:** Baseline characteristics by three tertile of sTfR index.

Characteristics	Overall (*N* = 11,525)	Tertile 1 (*N* = 3,842)	Tertile 2 (*N* = 3,841)	Tertile 3 (*N* = 3,842)	*p*-value
sTfR index	0.27–7.39	0.27–0.63	0.64–0.94	0.94–7.39	—
**Demographic**					
Age, year	38 [27, 48]	43 [31, 57]	37 [26, 48]	35 [24, 44]	<0.001
Gender, female (%)	9,147 (79.4)	2,445 (63.6)	3,151 (82.0)	3,551 (92.4)	<0.001
Ethnicity (%)					<0.001
Mexican American	2068 (17.9)	678 (17.6)	670 (17.4)	720 (18.7)	
Other Hispanic	1,110 (9.6)	383 (10.0)	347 (9.0)	380 (9.9)	
Non-Hispanic White	4,344 (37.7)	1,600 (41.6)	1,505 (39.2)	1,239 (32.2)	
Non-Hispanic Black	2,562 (22.2)	569 (14.8)	860 (22.4)	1,133 (29.5)	
Other Race	1,441 (12.5)	612 (15.9)	459 (12.0)	370 (9.6)	
Education (%)					<0.001
Less than high school	2,188 (19.0)	781 (20.3)	698 (18.2)	709 (18.5)	
High school or equivalent	2,325 (20.2)	809 (21.1)	756 (19.7)	760 (19.8)	
College or above	5,979 (51.9)	2074 (54.0)	2016 (52.5)	1889 (49.2)	
Missing	1,033 (9.0)	178 (4.6)	371 (9.7)	484 (12.6)	
BMI, kg/m^2^					<0.001
<18.5	274 (2.4)	92 (2.4)	97 (2.5)	85 (2.2)	
18.5–24.9	3,440 (29.8)	1,155 (30.1)	1,222 (31.8)	1,063 (27.7)	
25.0–29.9	3,243 (28.1)	1,196 (31.1)	1,047 (27.3)	1,000 (26.0)	
30.0–34.9	2,177 (18.9)	739 (19.2)	704 (18.3)	734 (19.1)	
≥35.0	2,250 (19.5)	614 (16.0)	726 (18.9)	910 (23.7)	
Missing	141 (1.2)	46 (1.2)	45 (1.2)	50 (1.3)	
Smoking status (%)					<0.001
Never/past	1872 (16.2)	767 (20.0)	622 (16.2)	483 (12.6)	
Current	2,199 (19.1)	883 (23.0)	687 (17.9)	629 (16.4)	
Missing	7,454 (64.7)	2,192 (57.1)	2,532 (65.9)	2,730 (71.1)	
Drinking status (%)					<0.001
Nondrinker	2,371 (20.6)	793 (20.6)	819 (21.3)	759 (19.8)	
Drinker	4,450 (38.6)	1778 (46.3)	1,434 (37.3)	1,238 (32.2)	
Missing	4,704 (40.8)	1,271 (33.1)	1,588 (41.3)	1845 (48.0)	
**Laboratory**					
sTfR, mg/L	3.10 [2.57, 3.94]	2.41 [2.11, 2.73]	3.10 [2.80, 3.50]	4.30 [3.70, 5.26]	<0.001
Ferritin, μg/L	57 [28, 116]	124 [75.10, 210]	58.60 [38, 95]	22 [12, 36.90]	<0.001
Albumin, g/dL	4.10 [3.90, 4.40]	4.20 [3.90, 4.40]	4.20 [3.90, 4.40]	4.10 [3.90, 4.30]	<0.001
Total cholesterol, mg/dL	184 [160, 211]	187 [161, 214]	183 [160, 211.50]	182 [159, 208]	<0.001
Serum creatinine, μmoI/L	67.18 [58.34, 78.68]	69.84 [59.23, 82.21]	67.18 [58.34, 79.56]	63.65 [56.58, 73.37]	<0.001
Serum potassium, mmol/L	3.90 [3.73, 4.20]	4 [3.80, 4.20]	3.90 [3.71, 4.17]	3.90 [3.70, 4.10]	<0.001
Hemoglobin, mg/dL	13.60 [12.80, 14.40]	14.10 [13.30, 15]	13.70 [13, 14.43]	13 [12.20, 13.80]	<0.001
MCV, f/L	88.70 [85.10, 91.90]	90.10 [87.30, 92.90]	89.20 [86.10, 92.10]	86.20 [81.80, 89.90]	<0.001
**Comorbidity (%)**					
Diabetes	1,011 (8.8)	377 (9.8)	322 (8.4)	312 (8.1)	0.019
Hypertension	2,798 (24.3)	1,043 (27.1)	906 (23.6)	849 (22.1)	<0.001
CVD	743 (6.4)	263 (6.8)	256 (6.7)	224 (5.8)	0.154
Cancer	728 (6.3)	277 (7.2)	265 (6.9)	186 (4.8)	<0.001
**Outcomes**					
All-cause mortality (%)	231 (2.0)	70 (1.8)	65 (1.7)	96 (2.5)	0.026
CVD mortality (%)	53 (0.5)	15 (0.4)	14 (0.4)	24 (0.6)	0.744
Cancer mortality (%)	60 (0.5)	17 (0.4)	17 (0.4)	26 (0.7)	0.002
Follow-up, months	51 [27, 145]	33 [23, 116]	55 [26, 149]	123 [35, 163]	<0.001

CVD, cardiovascular diseases; MCV, mean corpuscular volume.

### 3.2 Association of sTfR index with all-cause and cause-specific mortality

**Figure 2:**
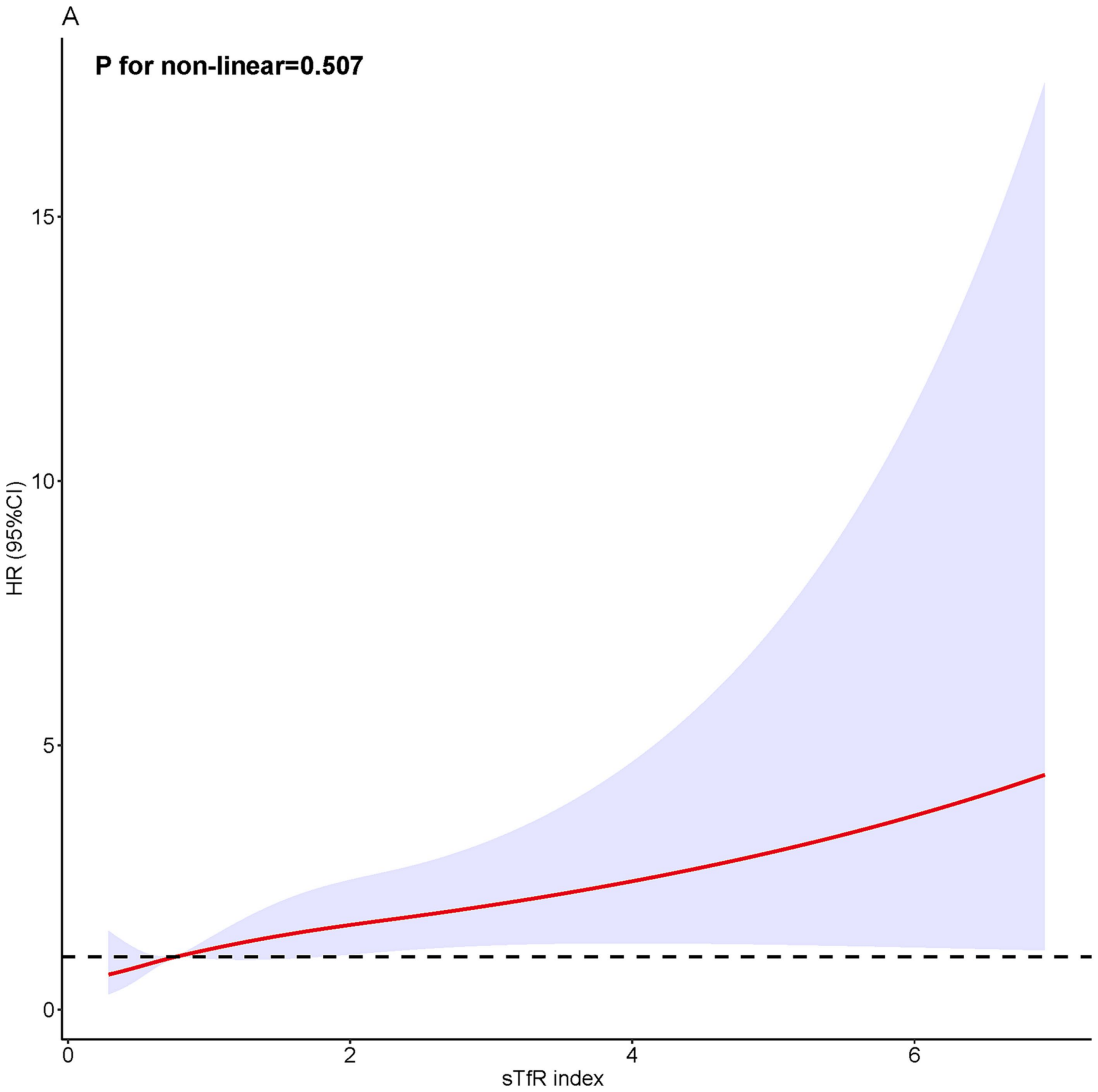
Restricted cubic spline analysis.

During a median follow-up of 51 months, 231 (2.0%) all-cause deaths occurred, including 53 (0.5%) CVD deaths and 60 (0.5%) cancer-related deaths. The risk of all-cause mortality, CVD-related mortality, and cancer-related mortality was higher in patients with higher sTfR index (Table 1 and Figure 2). After adjusting for confounders (Table 2), the HR of all-cause and cancer-related mortality associated with each SD increment in sTfR index was 1.25 [95% confidence interval (CI), 1.08–1.45] and 1.38 (1.07–1.77). Compared to participants in the lowest tertile, those in highest tertile of sTfR index had a 71% higher risk of all-cause mortality [hazard ratio (HR), 1.71, 95% CI, 1.14–2.57] after adjustment for all potential covariates (Table 2). A similar trend toward an increased risk of cancer-related mortality was observed. However, no significant association was found between sTfR index and CVD mortality (HR, 1.18; 95% CI, 0.83–1.68). There were approximately linear associations between sTfR index with all-cause mortality (*p* for non-linear association >0.05, Figure 2). That is the risk of all-cause mortality increased linearly as sTfR index increased. Regarding the mortality within 1 year (Supplementary Table S3), only 62 (0.5%) participants died and no significant association was found between the sTfR index and mortality. However, when considering the 10 years mortality, participants in the highest tertile of sTfR index were significantly associated with a higher risk of all-cause mortality compared to those in the lowest tertile (HR, 1.30; 95% CI, 1.11–1.53). Moreover, we performed separate analyses of patients with CVD or cancer at baseline and similar associations between sTfR index and mortality were showed (Table 3).

**Table 2:** The association between sTfR index and all-cause, CVD, and cancer mortality.

	All-cause mortality			CVD-related mortality			Cancer-related mortality		
	No. event	HR (95% CI)	*p*-value	No. event	sHR (95% CI)^a^	*p*-value	No. event	sHR (95% CI)^a^	*p*-value
**Model 1**									
Per SD increment	231	1.19 (1.07–1.32)	0.001	53	1.17 (0.92–1.49)	0.21	60	1.22 (1.01–1.48)	0.043
Tertile 1	70	1.00 (Ref.)	—	15	1.00 (Ref.)	—	17	1.00 (Ref.)	—
Tertile 2	65	0.97 (0.69–1.37)	0.870	14	1.10 (0.53–2.30)	0.800	17	0.96 (0.49–1.89)	0.906
Tertile 3	96	1.62 (1.17–2.25)	0.004	24	2.46 (1.25–4.83)	0.012	26	1.60 (0.84–3.03)	0.157
**Model 2**									
Per SD increment	231	1.20 (1.07–1.34)	0.002	53	1.06 (0.79–1.41)	0.721	60	1.25 (1.03–1.51)	0.032
Tertile 1	70	1.00 (Ref.)	—	15	1.00 (Ref.)	—	17	1.00 (Ref.)	—
Tertile 2	65	0.96 (0.68–1.36)	0.816	14	1.02 (0.48–2.16)	0.966	17	1.00 (0.50–2.02)	0.992
Tertile 3	96	1.55 (1.11–2.16)	0.010	24	1.73 (0.86–3.51)	0.139	26	1.80 (0.93–3.48)	0.090
**Model 3**									
Per SD increment	231	1.25 (1.08–1.45)	0.003	53	1.18 (0.83–1.68)	0.375	60	1.38 (1.07–1.77)	0.018
Tertile 1	70	1.00 (Ref.)	—	15	1.00 (Ref.)	—	17	1.00 (Ref.)	—
Tertile 2	65	1.08 (0.74–1.57)	0.689	14	1.19 (0.54–2.64)	0.674	17	1.19 (0.56–2.53)	0.654
Tertile 3	96	1.71 (1.14–2.57)	0.011	24	2.48 (1.05–5.87)	0.053	26	2.29 (1.02–5.14)	0.053

Model 1: unadjusted; model 2: adjusted for age, sex, ethnicity, smoking status, drinking status, and education; and model 3: further adjusted for BMI, SBP, DBP, albumin, total cholesterol, serum creatinine, serum potassium, hemoglobin, MCV, diabetes, hypertension, CVD, cancer, and ferritin.

a Sub-distribution hazard ratio accounting for the competing event.

**Table 3:** The association between sTfR index and all-cause mortality among patients with CVD and cancer.

	All-cause mortality		CVD-related mortality		Cancer-related mortality	
	HR (95%CI)	*p*-value	sHR (95% CI)^a^	*p*-value	sHR (95% CI)^a^	*p*-value
**Patients with CVD (*N* = 743)**						
Model 1	1.20 (0.97–1.48)	0.103	1.27 (0.96–1.70)	0.113	1.35 (0.87–2.10)	0.272
Model 2	1.23 (0.95–1.58)	0.126	1.37 (0.92–2.03)	0.498	1.74 (0.93–3.25)	0.475
Model 3	1.27 (0.91–1.77)	0.168	1.53 (0.96–2.42)	0.468	1.41 (0.77–2.56)	0.578
**Patients with cancer (*N* = 728)**						
Model 1	1.11 (0.83–1.49)	0.481	1.19 (0.73–1.95)	0.515	1.01 (0.54–1.87)	0.983
Model 2	1.00 (0.67–1.49)	0.994	0.48 (0.09–2.44)	0.631	1.32 (0.70–2.47)	0.638
Model 3	1.12 (0.67–1.88)	0.683	0.38 (0.07–2.13)	0.580	1.46 (0.48–4.48)	0.697

Model 1: unadjusted; model 2: adjusted for age, sex, ethnicity, smoking status, drinking status, and education; and model 3: further adjusted for BMI, SBP, DBP, albumin, total cholesterol, serum creatinine, serum potassium, hemoglobin, MCV, diabetes, hypertension, CVD, cancer, and ferritin.

a Sub-distribution hazard ratio accounting for the competing event.

**Figure 3:**
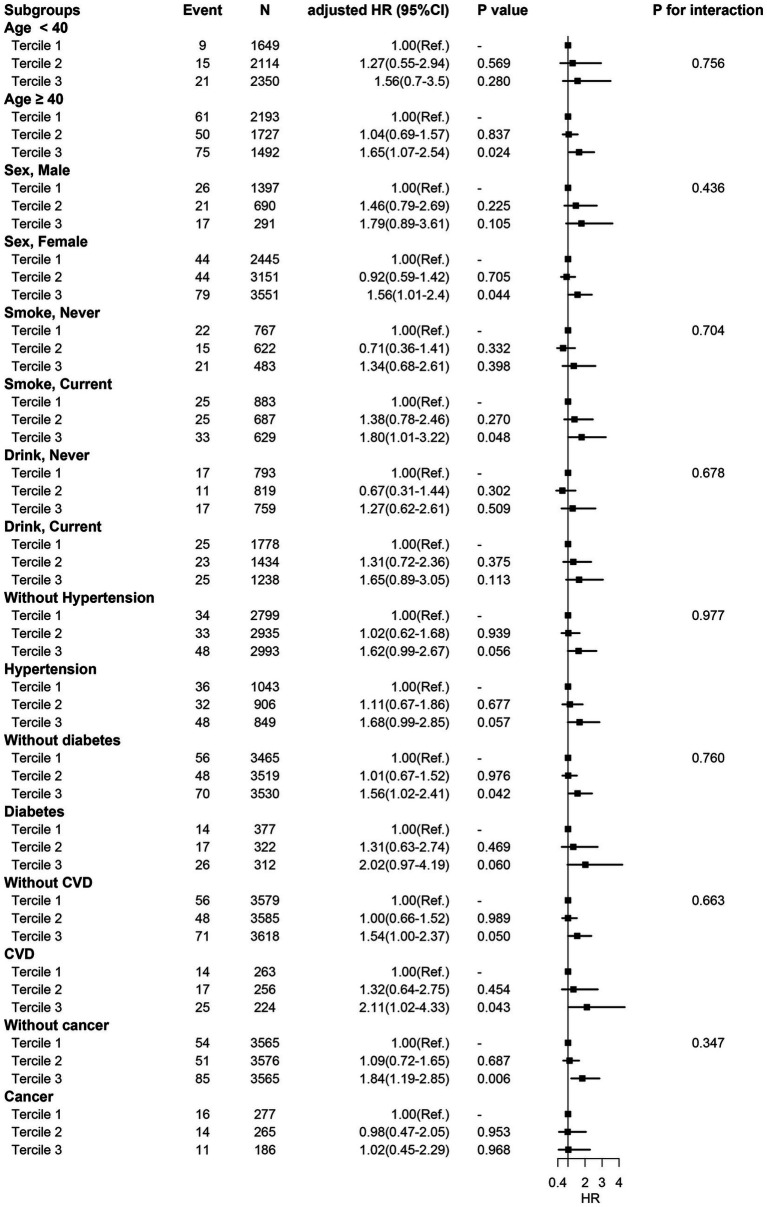
Subgroup analysis. HR was adjusted for age, sex, ethnicity, smoking status, drinking status, education, BMI, SBP, DBP, albumin, total cholesterol, serum creatinine, serum potassium, Hemoglobin, MCV, diabetes, hypertension, CVD, and ferritin.

In different subgroups of the population stratified by baseline characteristics and co-morbidities (Figure 3), the association between higher sTfR index and increased risk of all-cause mortality was consistent. Hence, baseline characteristics including age, sex, smoking status, hypertension, diabetes, CVD, and cancer did not affect the relationship between sTfR index and mortality (all *p* for interaction >0.05).

For additional analysis (Table 4), the HR for all-cause mortality, CVD-related mortality, and cancer-related mortality across three tertiles of ferritin were 1.09 (0.84–1.43), 1.00 (0.55–1.83), and 0.71 (0.44–1.15), respectively. Similarly, participants with iron deficiency (≤15 μg/L) were not significantly associated with study outcome as compared with those with normal levels of ferritin (Table 5), indicating that there was no significant association between ferritin levels and all-cause mortality, CVD-related mortality, and cancer-related mortality. In addition, we further excluded 1,326 patients who diagnosed with CVD or cancer at baseline to preclude the reverse causality, and consistent associations were found between sTfR index and study outcomes (Supplementary Table S4). For missing values, we excluded patients with missing values at baseline characteristics and repeated the analysis. The results of the sensitivity analysis were consistent to the primary analysis (Supplementary Table S5).

**Table 4:** The association between three tertiles of ferritin and all-cause, CVD, and cancer mortality.

	All-cause mortality			CVD-related mortality			Cancer-related mortality		
	No. event	HR (95% CI)	*p*-value	No. event	sHR (95% CI)^a^	*p*-value	No. event	sHR (95% CI)^a^	*p*-value
**Model 1**									
Per SD increment	231	1.02 (0.88–1.18)	0.793	53	1.04 (0.77–1.41)	0.804	60	0.94 (0.71–1.24)	0.667
Tertile 1	53	1.00 (Ref.)	—	10	1.00 (Ref.)	—	17	1.00 (Ref.)	—
Tertile 2	78	1.24 (0.87–1.77)	0.228	19	1.36 (0.63–2.97)	0.438	16	0.83 (0.42–1.65)	0.601
Tertile 3	100	1.29 (0.89–1.87)	0.173	24	1.09 (0.49–2.44)	0.839	27	1.27 (0.65–2.49)	0.482
**Model 2**									
Per SD increment	231	1 (0.87–1.16)	0.961	53	1.09 (0.8–1.49)	0.581	60	0.89 (0.68–1.18)	0.439
Tertile 1	53	1.00 (Ref.)	—	10	1.00 (Ref.)	—	17	1.00 (Ref.)	—
Tertile 2	78	1.14 (0.8–1.63)	0.477	19	1.24 (0.56–2.74)	0.595	16	0.82 (0.41–1.64)	0.574
Tertile 3	100	1.2 (0.83–1.73)	0.334	24	1.15 (0.52–2.57)	0.727	27	1.1 (0.56–2.17)	0.781
**Model 3**									
Per SD increment	231	1.09 (0.84–1.43)	0.519	53	1.00 (0.55–1.83)	0.989	60	0.71 (0.44–1.15)	0.178
Tertile 1	53	1.00 (Ref.)	—	10	1.00 (Ref.)	—	17	1.00 (Ref.)	—
Tertile 2	78	1.32 (0.91–1.92)	0.15	19	1.19 (0.52–2.70)	0.681	16	0.9 (0.43–1.87)	0.781
Tertile 3	100	1.55 (0.96–2.51)	0.077	24	0.94 (0.33–2.65)	0.907	27	1.33 (0.54–3.32)	0.540

Model 1: unadjusted; model 2: adjusted for age, sex, ethnicity, smoking status, drinking status, and education; and model 3: further adjusted for BMI, SBP, DBP, albumin, total cholesterol, serum creatinine, serum potassium, Hemoglobin, MCV, diabetes, hypertension, CVD, cancer, and ferritin.

a Sub-distribution hazard ratio accounting for the competing event.

**Table 5:** The association between iron deficiency and all-cause, CVD, and cancer mortality.^a^

	All-cause mortality		CVD-related mortality		Cancer-related mortality	
	HR (95% CI)	*p*-value	sHR (95% CI)^b^	*p*-value	sHR (95% CI)^b^	*p*-value
Model 1	1.20 (0.77–1.85)	0.424	0.83 (0.25–2.74)	0.758	1.71 (0.82–3.56)	0.16
Model 2	1.24 (0.80–1.92)	0.341	0.86 (0.26–2.84)	0.801	1.74 (0.83–3.65)	0.151
Model 3	1.16 (0.74–1.82)	0.528	0.80 (0.24–2.74)	0.731	1.69 (0.80–3.57)	0.179

Model 1: unadjusted; model 2: adjusted for age, sex, ethnicity, smoking status, drinking status, and education; and model 3: further adjusted for BMI, SBP, DBP, albumin, total cholesterol, serum creatinine, serum potassium, Hemoglobin, MCV, diabetes, hypertension, CVD, and cancer.

a Iron deficiency was defined as serum ferritin ≤15 μg/L.

b Sub-distribution hazard ratio accounting for the competing event.

## 4 Discussion

Based on a nationally representative sample of US participants, this study found that sTfR index was independently associated with an increased risk of all-cause death and cancer related death, regardless of ferritin levels. After adjusting for confounding factors, each SD increment in sTfR index was associated with a 25% increasing risk of all-cause death. The association between sTfR index and all-cause mortality remained consistent in different subgroups stratified by age, sex, smoking status, hypertension, diabetes, and CVD. However, ferritin was not a predictor of all-cause death after adjusting for confounding factors. This study might provide additional insights into the implications of the sTfR index in assessing mortality risk and future research directions of investigating the underlying pathways linking chronic inflammation, functional iron deficiency, and adverse health outcomes, which could provide valuable insights into novel therapeutic targets and interventions.

A growing body of evidence suggests that iron imbalance plays a crucial role in various subtypes of cardiovascular disease, including atherosclerosis, drug-induced heart failure, myocardial ischaemia-reperfusion injury, sepsis-induced cardiomyopathy, arrhythmia and diabetic cardiomyopathy (1, 19). Some studies have demonstrated that increased sTfR levels are associated with higher mortality in patients with heart failure (19, 27–30). High serum sTfR accurately reflect depleted iron stores in the bone marrow of heart failure patients and identify those at higher risk of 3 years mortality (29). However, in this study, after adjusting for variables, there was no significant correlation between sTfR index and CVD-related mortality.

The main reasons for this lack of significant correlation may be attributed to several factors. First, the number of CVD-related deaths in this cohort was relatively small, which could potentially affect statistical efficiency. Second, the complexity of the CVD population in this study, which includes congestive heart failure, coronary heart disease, angina pectoris, heart attack, or stroke, might have implications on the results. However, the mortality rate of heart failure patients could not be separately obtained, which could lead to differences in the outcome.

Iron (Fe) has been indicated to play a critical role in leukemia cell growth (31). The expression of soluble transferrin receptor (sTfR) has also been identified in many malignant tumours (2). In lung cancer, lymphoma, and breast cancer, the expression of sTfR has been shown to correlate with tumor differentiation, suggesting a potential prognostic value (22). Consistent with these findings, our study also demonstrated that each SD increment in sTfR index was associated with a 38% increased risk of cancer-related death.

Several reasons may explain why sTfR index increment, independent of ferritin levels, is associated with an increased risk of all-cause death. First, sTfR index is valuable in diagnosing iron-deficiency anemia (1), and anemia itself reflects a person’s overall health status and disease severity (32). Second, iron deficiency is a health-related condition in which iron availability is insufficient to meet the body’s needs and which also can be present without anemia (33–35). Iron deficiency in patients with chronic heart failure can worsen the underlying condition and negatively impact clinical outcomes and quality of life (33, 34). Iron deficiency is also common in patients with idiopathic pulmonary arterial hypertension and is associated with disease severity and poor clinical outcome (35). Third, a confirmed relationship exists between reduced iron concentration and the occurrence of frailty syndrome (36). Patients with diagnosed frailty syndrome represent a unique group of individuals with chronic illnesses. In the classic definition, frailty syndrome includes parameters such as reduced muscle strength, subjective fatigue, unintentional weight loss, slow gait, and low physical activity, which increase the incidence of adverse events, such as falls, hospitalizations and even death (36). Moreover, iron deficiency commonly co-occurs with depressive symptoms in older individuals (21). Lastly, elevated sTfR levels are characteristic of functional iron deficiency, a condition defined by tissue iron deficiency despite adequate iron stores (2).

Furthermore, sTfR may have other functions beyond detecting iron deficiency that merit further investigation. First, apart from erythrocytes, activated lymphocytes also release a soluble form of the human transferrin receptor *in vitro* (37). Second, iron deficiency frequently co-occurs with chronic inflammatory diseases (34). The sTfR has been found to significantly and positively correlate with CRP concentration (36) and antioxidant status, independently of covariates such as serum ferritin and hepcidin (38). Mild elevation of sTfR levels in multiple sclerosis patients may indicate active inflammation with ongoing oxidative damage that is not detectable through history or examination (39). Third, the frequency of the G allele at the position 210 of the transferrin receptor gene was significantly higher in type 2 diabetes patients (40). The sTfR levels could be spuriously elevated in subjects with insulin resistance (41). Both insulin sensitivity and glucose tolerance status are significantly associated with serum sTfR concentrations, with insulin sensitivity mainly predicts circulating sTfR in subjects with normal glucose tolerance (NGT). The implications of the interrelationships between iron and glucose metabolism warrant further investigation (42). Finally, patients with *H. pylori* infection showed higher sTfR concentration and higher sTfR index levels (43).

Several limitations of this study should be noted in this study. First, like all retrospective studies, there may be other potential and unknown confounders that were not considered. However, we made efforts to adjust for as many confounders as possible and achieved good balance in the PSM cohorts. Second, given the established link between inflammation, iron metabolism, and mortality outcomes, it may not be fully considered that inflammation markers were not included as covariates in the analysis. Third, the relatively small number of CVD-related and cancer-related deaths in the cohort could potentially affect statistical efficiency to some extent. Fourth, as the data we analyzed was obtained from an observational database, the results reported in our study need further validation through additional randomized trials. Fifth, although the median follow-up was 51 months, the shortest follow-up duration is less than 1 year, therefore, the accuracy of the relationship between sTfR index and death needs to be interpreted with caution.

In conclusion, our study revealed that higher sTfR index was significantly and linearly associated with higher risks of all-cause and cancer-related mortality. Adding sTfR index to assessments of overall health may identify more individuals at risk for mortality and thus has the potential to improve decisions to implement preventative or treatment approaches.

## Data availability statement

The original contributions presented in the study are included in the article/Supplementary material, further inquiries can be directed to the corresponding authors.

## Ethics statement

The studies involving human participants were reviewed and approved by the NHANES Research Ethics Review Committee granted approval for the NHANES research protocols for 2003–2020, with all participants providing written informed consent. The patients/participants provided their written informed consent to participate in this study.

## Author contributions

YY: Conceptualization, Data curation, Investigation, Writing – original draft. DL: Formal analysis, Methodology, Software, Writing – original draft. ZZ: Funding acquisition, Project administration, Resources, Visualization, Writing – review & editing. LT: Project administration, Supervision, Validation, Writing – original draft, Writing – review & editing.

## References

[1] Speeckaert MM Speeckaert R Delanghe JR. Biological and clinical aspects of soluble transferrin receptor. Crit Rev Clin Lab Sci. (2010) 47:213–28. doi: 10.3109/10408363.2010.550461, PMID: 21391831

[2] Beguin Y. Soluble transferrin receptor for the evaluation of erythropoiesis and iron status. Clin Chim Acta. (2003) 329:9–22. doi: 10.1016/s0009-8981(03)00005-6 12589962

[3] Arosio P Levi S. Cytosolic and mitochondrial ferritins in the regulation of cellular iron homeostasis and oxidative damage. Biochim Biophys Acta. (2010) 1800:783–92. doi: 10.1016/j.bbagen.2010.02.005, PMID: 20176086

[4] Truman-Rosentsvit M Berenbaum D Spektor L Cohen LA Belizowsky-Moshe S Lifshitz L. Ferritin is secreted via 2 distinct nonclassical vesicular pathways. Blood. (2018) 131:342–52. doi: 10.1182/blood-2017-02-768580, PMID: 29074498 PMC5774206

[5] Annous Y Manning S Khoujah D. Ferritin, fever, and frequent visits: hyperferritinemic syndromes in the emergency department. Am J Emerg Med. (2021) 48:249–54. doi: 10.1016/j.ajem.2021.04.088, PMID: 34000525

[6] Lehmberg K McClain KL Janka GE Allen CE. Determination of an appropriate cut-off value for ferritin in the diagnosis of hemophagocytic lymphohistiocytosis. Pediatr Blood Cancer. (2014) 61:2101–3. doi: 10.1002/pbc.25058, PMID: 24753034

[7] Skikne BS Punnonen K Caldron PH Bennett MT Rehu M Gasior GH. Improved differential diagnosis of anemia of chronic disease and iron deficiency anemia: a prospective multicenter evaluation of soluble transferrin receptor and the sTfR/log ferritin index. Am J Hematol. (2011) 86:923–7. doi: 10.1002/ajh.22108, PMID: 21812017

[8] Pfeiffer CM Looker AC. Laboratory methodologies for indicators of iron status: strengths, limitations, and analytical challenges. Am J Clin Nutr. (2017) 106:1606s–14s. doi: 10.3945/ajcn.117.155887, PMID: 29070545 PMC5701713

[9] Skikne BS Flowers CH Cook JD. Serum transferrin receptor: a quantitative measure of tissue iron deficiency. Blood. (1990) 75:1870–6. doi: 10.1182/blood.V75.9.1870.1870, PMID: 2331526

[10] Cook JD Flowers CH Skikne BS. The quantitative assessment of body iron. Blood. (2003) 101:3359–63. doi: 10.1182/blood-2002-10-3071 12521995

[11] Ronnenberg AG Wood RJ Wang X Xing H Chen C Chen D. Preconception hemoglobin and ferritin concentrations are associated with pregnancy outcome in a prospective cohort of Chinese women. J Nutr. (2004) 134:2586–91. doi: 10.1093/jn/134.10.2586, PMID: 15465752

[12] Grant FK Martorell R Flores-Ayala R Cole CR Ruth LJ Ramakrishnan U. Comparison of indicators of iron deficiency in Kenyan children. Am J Clin Nutr. (2012) 95:1231–7. doi: 10.3945/ajcn.111.029900, PMID: 22456661 PMC4697948

[13] Punnonen K Irjala K Rajamäki A. Serum transferrin receptor and its ratio to serum ferritin in the diagnosis of iron deficiency. Blood. (1997) 89:1052–7. doi: 10.1182/blood.V89.3.1052 9028338

[14] Weiss G Goodnough LT. Anemia of chronic disease. N Engl J Med. (2005) 352:1011–23. doi: 10.1056/NEJMra041809 15758012

[15] Skikne BS. Serum transferrin receptor. Am J Hematol. (2008) 83:872–5. doi: 10.1002/ajh.21279 18821709

[16] Oustamanolakis P Koutroubakis IE. Soluble transferrin receptor-ferritin index is the most efficient marker for the diagnosis of iron deficiency anemia in patients with IBD. Inflamm Bowel Dis. (2011) 17:E158–9. doi: 10.1002/ibd.21881, PMID: 21953900

[17] Krawiec P Pac-Kożuchowska E. Soluble transferrin receptor and soluble transferrin receptor/log ferritin index in diagnosis of iron deficiency anemia in pediatric inflammatory bowel disease. Dig Liver Dis. (2019) 51:352–7. doi: 10.1016/j.dld.2018.11.012, PMID: 30538074

[18] Suominen P Punnonen K Rajamäki A Irjala K. Serum transferrin receptor and transferrin receptor-ferritin index identify healthy subjects with subclinical iron deficits. Blood. (1998) 92:2934–9. doi: 10.1182/blood.V92.8.2934.420k07_2934_2939, PMID: 9763580

[19] Fang X Ardehali H Min J Wang F. The molecular and metabolic landscape of iron and ferroptosis in cardiovascular disease. Nat Rev Cardiol. (2022) 20:7–23. doi: 10.1038/s41569-022-00735-4, PMID: 35788564 PMC9252571

[20] Zhu S Liu C Zhao C Chen G Meng S Hong M. Increased serum soluble transferrin receptor levels were associated with high prevalence of cardiovascular diseases: insights from the National Health and Nutrition Examination Survey 2017–2018. Front Cell Dev Biol. (2022) 10:874846. doi: 10.3389/fcell.2022.874846, PMID: 35493097 PMC9039157

[21] Zhang Y Xue N Jia W Chen X Chen X Li H. Associations between serum soluble transferrin receptor and the prevalence of cancers. Front Oncol. (2022) 12:1039930. doi: 10.3389/fonc.2022.1039930, PMID: 36568176 PMC9773974

[22] Dowlati A Loo M Bury T Fillet G Beguin Y. Soluble and cell-associated transferrin receptor in lung cancer. Br J Cancer. (1997) 75:1802–6. doi: 10.1038/bjc.1997.307, PMID: 9192985 PMC2223608

[23] Fernández-Cao JC Arija V Aranda N Basora J Diez-Espino J Estruch R. Soluble transferrin receptor and risk of type 2 diabetes in the obese and nonobese. Eur J Clin Investig. (2017) 47:221–30. doi: 10.1111/eci.12725, PMID: 28075490

[24] Wang H Qi Q Song S Zhang D Feng L. Association between soluble transferrin receptor and systolic hypertension in adults: National Health and Nutrition Examination Survey (2007–2010 and 2015–2018). Front Cardiovasc Med. (2022) 9:1029714. doi: 10.3389/fcvm.2022.1029714, PMID: 36407469 PMC9671951

[25] Jiang Y Jiang FQ Kong F An MM Jin BB Cao D. Inflammatory anemia-associated parameters are related to 28-day mortality in patients with sepsis admitted to the ICU: a preliminary observational study. Ann Intensive Care. (2019) 9:67. doi: 10.1186/s13613-019-0542-7, PMID: 31183575 PMC6557959

[26] Gao M Piernas C Astbury NM Hippisley-Cox J O’Rahilly S Aveyard P. Associations between body-mass index and COVID-19 severity in 6·9 million people in England: a prospective, community-based, cohort study. Lancet Diabetes Endocrinol. (2021) 9:350–9. doi: 10.1016/s2213-8587(21)00089-9, PMID: 33932335 PMC8081400

[27] Dhaliwal S Kalogeropoulos AP. Markers of iron metabolism and outcomes in patients with heart failure: a systematic review. Int J Mol Sci. (2023) 24:5645. doi: 10.3390/ijms24065645, PMID: 36982717 PMC10059277

[28] Grammer TB Scharnagl H Dressel A Kleber ME Silbernagel G Pilz S. Iron metabolism, hepcidin, and mortality (the Ludwigshafen risk and cardiovascular health study). Clin Chem. (2019) 65:849–61. doi: 10.1373/clinchem.2018.297242, PMID: 30917972

[29] Sierpinski R Josiak K Suchocki T Wojtas-Polc K Mazur G Butrym A. High soluble transferrin receptor in patients with heart failure: a measure of iron deficiency and a strong predictor of mortality. Eur J Heart Fail. (2021) 23:919–32. doi: 10.1002/ejhf.2036, PMID: 33111457

[30] Weidmann H Bannasch JH Waldeyer C Shrivastava A Appelbaum S Ojeda-Echevarria FM. Iron metabolism contributes to prognosis in coronary artery disease: prognostic value of the soluble transferrin receptor within the AtheroGgene study. J Am Heart Assoc. (2020) 9:e015480. doi: 10.1161/jaha.119.015480, PMID: 32321351 PMC7428563

[31] Taetle R Rhyner K Castagnola J To D Mendelsohn J. Role of transferrin, Fe, and transferrin receptors in myeloid leukemia cell growth. Studies with an antitransferrin receptor monoclonal antibody. J Clin Invest. (1985) 75:1061–7. doi: 10.1172/jci111768, PMID: 2984253 PMC423664

[32] Corazza F Beguin Y Bergmann P André M Ferster A Devalck C. Anemia in children with cancer is associated with decreased erythropoietic activity and not with inadequate erythropoietin production. Blood. (1998) 92:1793–8. doi: 10.1182/blood.V92.5.1793, PMID: 9716610

[33] Lam CSP Doehner W Comin-Colet J. Iron deficiency in chronic heart failure: case-based practical guidance. ESC Heart Fail. (2018) 5:764–71. doi: 10.1002/ehf2.12333, PMID: 30073785 PMC6165963

[34] Cappellini MD Comin-Colet J de Francisco A Dignass A Doehner W Lam CS. Iron deficiency across chronic inflammatory conditions: international expert opinion on definition, diagnosis, and management. Am J Hematol. (2017) 92:1068–78. doi: 10.1002/ajh.24820, PMID: 28612425 PMC5599965

[35] Rhodes CJ Howard LS Busbridge M Ashby D Kondili E Gibbs JSR. Iron deficiency and raised hepcidin in idiopathic pulmonary arterial hypertension: clinical prevalence, outcomes, and mechanistic insights. J Am Coll Cardiol. (2011) 58:300–9. doi: 10.1016/j.jacc.2011.02.057, PMID: 21737024

[36] Zawadzki B Mazur G Butrym A. Iron dysregulation and frailty syndrome. J Clin Med. (2021) 10:5596. doi: 10.3390/jcm10235596, PMID: 34884301 PMC8658196

[37] Woith W Nüsslein I Antoni C Dejica DI Winkler TH Herrmann M. A soluble form of the human transferrin receptor is released by activated lymphocytes *in vitro*. Clin Exp Immunol. (1993) 92:537–42. doi: 10.1111/j.1365-2249.1993.tb03434.x, PMID: 8513587 PMC1554787

[38] Suárez-Ortegón MF Arbeláez A Moreno-Navarrete JM Ortega-Ávila JG Mosquera M Fernández-Real JM. Soluble transferrin receptor, antioxidant status and cardiometabolic risk in apparently healthy individuals. Antioxidants. (2022) 12. doi: 10.3390/antiox12010019, PMID: 36670881 PMC9854855

[39] Sfagos C Makis AC Chaidos A Hatzimichael EC Dalamaga A Kosma K. Serum ferritin, transferrin and soluble transferrin receptor levels in multiple sclerosis patients. Mult Scler. (2005) 11:272–5. doi: 10.1191/1352458505ms1171oa, PMID: 15957506

[40] Fernández-Real JM Mercader JM Ortega FJ Moreno-Navarrete JM López-Romero P Ricart W. Transferrin receptor-1 gene polymorphisms are associated with type 2 diabetes. Eur J Clin Investig. (2010) 40:600–7. doi: 10.1111/j.1365-2362.2010.02306.x, PMID: 20497464

[41] Suárez-Ortegón MF McLachlan S Wild SH Fernández-Real JM Hayward C Polašek O. Soluble transferrin receptor levels are positively associated with insulin resistance but not with the metabolic syndrome or its individual components. Br J Nutr. (2016) 116:1165–74. doi: 10.1017/s0007114516002968, PMID: 27605239 PMC5860738

[42] Fernández-Real JM Moreno JM López-Bermejo A Chico B Vendrell J Ricart W. Circulating soluble transferrin receptor according to glucose tolerance status and insulin sensitivity. Diabetes Care. (2007) 30:604–8. doi: 10.2337/dc06-1138, PMID: 17327328

[43] Enko D Wagner H Kriegshäuser G Wögerer J Halwachs-Baumann G Schnedl WJ. Iron status determination in individuals with *Helicobacter pylori* infection: conventional vs. new laboratory biomarkers. Clin Chem Lab Med. (2019) 57:982–9. doi: 10.1515/cclm-2018-1182, PMID: 31154451

