# Supplementary material for: Association of soluble transferrin receptor/log ferritin index with all-cause and cause-specific mortality: National Health and Nutrition Examination Survey
Source: Front Nutr. 2024 Feb 27;11:1275522. doi: 10.3389/fnut.2024.1275522 (PMC10927731; doi:10.3389/fnut.2024.1275522)
Supplement: Supplementary file 1 [file Data_Sheet_1.docx]

**Supplemental Table S1. Baseline characteristics among participants with and without serum ferritin measurement**

| **Characteristics** | **Without ferritin measurement (N=28735)** | **With ferritin measurement**  **(N=41783)** |
| --- | --- | --- |
| **Demographic** |  |  |
| Age, yr | 54[36,67] | 38[27,48] |
| Gender, female (%) | 11033(38.4) | 9593(79.3) |
| Ethnicity (%) |  |  |
| Mexican American | 4908(17.1) | 2193(18.1) |
| Other Hispanic | 2370(8.2) | 1155(9.5) |
| Non-Hispanic White | 13024(45.3) | 4532(37.5) |
| Non-Hispanic Black | 5959(20.7) | 2712(22.4) |
| Other Race | 2477(8.6) | 1504(12.4) |
| Education (%) |  |  |
| Less than high school | 7663(26.7) | 2330(19.3) |
| High school or equivalent | 6549(22.8) | 2448(20.2) |
| College or above | 12928(45.0) | 6247(51.6) |
| Missing | 1598(5.6) | 1071(8.9) |
| BMI, kg/m^2^ |  |  |
| <18.5 | 418(1.5) | 292(2.4) |
| 18.5–24.9 | 7451(25.9) | 3645(30.1) |
| 25.0–29.9 | 9222(32.1) | 3391(28.0) |
| 30.0-34.9 | 5585(19.4) | 2290(18.9) |
| ≥35.0 | 3926(13.7) | 2324(19.2) |
| Missing | 2136(7.4) | 154(1.3) |
| Smoking status (%) |  |  |
| Never/past | 7488(26.1) | 1952(16.1) |
| Current | 5805(20.2) | 2334(19.3) |
| Missing | 15445(53.7) | 7810(64.6) |
| Drinking status (%) |  |  |
| Nondrinker | 5373(18.7) | 2490(20.6) |
| Drinker | 9943(34.6) | 4662(38.5) |
| Missing | 13422(46.7) | 4944(40.9) |
| **Laboratory** |  |  |
| sTfR,mg/l | 3.84[2.98,6.37] | 3.10[2.54,4] |
| Ferritin, μg/L | - | 57[27,118] |
| Albumin, g/dl | 4.30[4.10,4.50] | 4.10[3.90,4.40] |
| Total cholesterol, mg/dl | 192[165,222] | 184[160,211] |
| Serum creatinine, μmoI/L | 79.56[69.84,93.70] | 66.30[57.46,78.68] |
| Serum potassium, mmol/L | 4[3.80,4.20] | 3.90[3.72,4.20] |
| Hemoglobin, mg/dL | 14.50[13.50,15.50] | 13.60[12.70,14.40] |
| MCV, f/L | 89.90[86.80,93.10] | 88.60[84.90,91.90] |
| **Comorbidity** (%) |  |  |
| Diabetes | 3906(13.6) | 1069(8.8) |
| Hypertension | 11066(38.5) | 2945(24.3) |
| CVD | 3762(13.1) | 779(6.4) |
| Cancer | 2988(10.4) | 767(6.3) |

CVD, cardiovascular diseases; MCV, Mean Corpuscular Volume

**Supplemental Table S2. Baseline characteristics among participants with and without death follow-up**

| **Characteristics** | **Without death follow-up (N=13151)** | **With death follow-up(N=41783)** |
| --- | --- | --- |
| **Demographic** |  |  |
| Age, yr | 39[17,60] | 47[31,64] |
| Gender, female (%) | 6665(50.7) | 21575(51.6) |
| Ethnicity (%) |  |  |
| Mexican American | 1955(14.9) | 7358(17.6) |
| Other Hispanic | 1249(9.5) | 3583(8.6) |
| Non-Hispanic White | 4367(33.2) | 17924(42.9) |
| Non-Hispanic Black | 3533(26.9) | 8854(21.2) |
| Other Race | 2047(15.6) | 4064(9.7) |
| Education (%) |  |  |
| Less than high school | 1779(13.5) | 10226(24.5) |
| High school or equivalent | 2245(17.1) | 9182(22.0) |
| College or above | 5305(40.3) | 19640(47.0) |
| Missing | 3822(29.1) | 2735(6.5) |
| BMI, kg/m^2^ |  |  |
| <18.5 | 382(2.9) | 723(1.7) |
| 18.5–24.9 | 3960(30.1) | 11342(27.1) |
| 25.0–29.9 | 3445(26.2) | 12913(30.9) |
| 30.0-34.9 | 2220(16.9) | 8071(19.3) |
| ≥35.0 | 2087(15.9) | 6412(15.3) |
| Missing | 1057(8.0) | 2322(5.6) |
| Smoking status (%) |  |  |
| Never/past | 2224(16.9) | 9615(23.0) |
| Current | 1697(12.9) | 8225(19.7) |
| Missing | 9230(70.2) | 23943(57.3) |
| Drinking status (%) |  |  |
| Nondrinker | 2146(16.3) | 8014(19.2) |
| Drinker | 3749(28.5) | 14896(35.7) |
| Missing | 7256(55.2) | 18873(45.2) |
| **Laboratory** |  |  |
| sTfR,mg/l | 3.13[2.58,3.98] | 3.10[2.55,4] |
| Ferritin, μg/L | 86.80[38.50,173] | 53.70[25.63,112] |
| Albumin, g/dl | 4.10[3.90,4.40] | 4.20[4,4.50] |
| Total cholesterol, mg/dl | 174[150,202] | 190[164,219] |
| Serum creatinine, μmoI/L | 72.49[61.88,85.75] | 75.14[61.88,88.40] |
| Serum potassium, mmol/L | 4.10[3.80,4.30] | 4[3.80,4.20] |
| Hemoglobin, mg/dL | 14[13,15.10] | 14.10[13.10,15.20] |
| MCV, f/L | 88.50[85.10,91.60] | 89.60[86.20,92.70] |
| **Comorbidity** (%) |  |  |
| Diabetes | 1455(11.1) | 4987(11.9) |
| Hypertension | 3707(28.2) | 14080(33.7) |
| CVD | 1182(9.0) | 4549(10.9) |
| Cancer | 1009(7.7) | 3769(9.0) |

CVD, cardiovascular diseases; MCV, Mean Corpuscular Volume

**Supplemental Table S3. The association between sTfR index and all-cause, CVD, and cancer mortality within 1, 5, and 10 years.**

|  | All-cause mortality | | CVD-related mortality | | Cancer-related mortality | |
| --- | --- | --- | --- | --- | --- | --- |
|  | HR (95%CI) | P value | sHR (95%CI) * | P value | sHR (95%CI) * | P value |
| 1 year |  |  |  |  |  |  |
| model 1 | 1.13(0.84-1.51) | 0.416 | 1.36(0.90-2.05) | 0.170 | 0.80(0.30-2.15) | 0.669 |
| model 2 | 0.98(0.67-1.43) | 0.909 | 1.17(0.62-2.20) | 0.767 | 0.63(0.22-1.81) | 0.637 |
| model 3 | 0.87(0.55-1.38) | 0.566 | 0.94(0.41-2.13) | 0.920 | 0.70(0.28-1.78) | 0.670 |
| 5 years |  |  |  |  |  |  |
| model 1 | 1.22(1.06-1.41) | 0.006 | 1.31(0.97-1.75) | 0.086 | 1.04(0.68-1.58) | 0.865 |
| model 2 | 1.21(1.03-1.42) | 0.021 | 1.13(0.74-1.72) | 0.58 | 1.04(0.66-1.64) | 0.883 |
| model 3 | 1.24(1.00-1.52) | 0.052 | 1.28(0.76-2.16) | 0.452 | 1.19(0.71-1.99) | 0.694 |
| 10 years |  |  |  |  |  |  |
| model 1 | 1.21(1.08-1.36) | 0.001 | 1.19(0.90-1.56) | 0.226 | 1.15(0.89-1.49) | 0.297 |
| model 2 | 1.22(1.08-1.38) | 0.002 | 1.05(0.74-1.48) | 0.798 | 1.18(0.9-1.53) | 0.241 |
| model 3 | 1.30(1.11-1.53) | 0.002 | 1.27(0.85-1.88) | 0.263 | 1.36(0.98-1.87) | 0.084 |

*: sub-distribution hazard ratio accounting for the competing event

Model 1: unadjusted; model 2: adjusted for age, sex, ethnicity, smoking status, drinking status, and education; and model 3: further adjusted for BMI, SBP, DBP, albumin, total cholesterol, serum creatinine, serum potassium, Hemoglobin, MCV, diabetes, hypertension, CVD, cancer, and ferritin

**Supplemental** **Table S4 the association between sTfR index and all-cause, CVD, and cancer mortality after excluding patients with CVD or cancer**

|  | All-cause mortality | | | CVD-related mortality | | | Cancer-related mortality | | |
| --- | --- | --- | --- | --- | --- | --- | --- | --- | --- |
|  | N. event | HR (95%CI) | P value | N. event | sHR (95%CI) * | P value | N. event | sHR (95%CI) * | P value |
| Model 1 |  |  |  |  |  |  |  |  |  |
| Per SD increment | 150 | 1.19(1.06-1.35) | 0.005 | 26 | 1.17(0.92-1.49) | 0.210 | 43 | 1.25(1.02-1.55) | 0.040 |
| tertile 1 | 44 | 1.00(Ref.) | - | 7 | 1.00(Ref.) | - | 13 | 1.00(Ref.) | - |
| tertile 2 | 39 | 0.85(0.55-1.32) | 0.465 | 6 | 1.1(0.53-2.3) | 0.800 | 10 | 0.69(0.30-1.60) | 0.397 |
| tertile 3 | 67 | 1.45(0.97-2.18) | 0.074 | 13 | 2.46(1.25-4.83) | 0.012 | 20 | 1.38(0.66-2.89) | 0.402 |
| Model 2 |  |  |  |  |  |  |  |  |  |
| Per SD increment | 231 | 1.21(1.07-1.37) | 0.003 | 26 | 1.06(0.79-1.41) | 0.721 | 43 | 1.24(1.01-1.54) | 0.055 |
| tertile 1 | 70 | 1.00(Ref.) | - | 7 | 1.00(Ref.) | - | 13 | 1.00(Ref.) | - |
| tertile 2 | 65 | 0.89(0.57-1.39) | 0.609 | 6 | 1.02(0.48-2.16) | 0.966 | 10 | 0.76(0.32-1.78) | 0.535 |
| tertile 3 | 96 | 1.57(1.04-2.38) | 0.034 | 13 | 1.73(0.86-3.51) | 0.139 | 20 | 1.54(0.71-3.32) | 0.291 |
| Model 3 |  |  |  |  |  |  |  |  |  |
| Per SD increment | 231 | 1.24(1.05-1.47) | 0.013 | 26 | 1.18(0.83-1.68) | 0.375 | 43 | 1.35(1.02-1.79) | 0.061 |
| tertile 1 | 70 | 1.00(Ref.) | - | 7 | 1.00(Ref.) | - | 13 | 1.00(Ref.) | - |
| tertile 2 | 65 | 1.01(0.62-1.63) | 0.974 | 6 | 1.19(0.54-2.64) | 0.674 | 10 | 0.87(0.35-2.20) | 0.780 |
| tertile 3 | 96 | 1.68(1.00-2.81) | 0.051 | 13 | 2.48(1.05-5.87) | 0.053 | 20 | 1.82(0.70-4.75) | 0.245 |

*: sub-distribution hazard ratio accounting for the competing event

Model 1: unadjusted; model 2: adjusted for age, sex, ethnicity, smoking status, drinking status, and education; and model 3: further adjusted for BMI, SBP, DBP, albumin, total cholesterol, serum creatinine, serum potassium, Hemoglobin, MCV, diabetes, hypertension, CVD, cancer, and ferritin

**Supplemental Table S5 the association between sTfR index and all-cause, CVD, and cancer mortality after** **eliminating patients with missing values**

|  | All-cause mortality | | | CVD-related mortality | | | Cancer-related mortality | | |
| --- | --- | --- | --- | --- | --- | --- | --- | --- | --- |
|  | N. event | HR (95%CI) | P value | N. event | sHR (95%CI) * | P value | N. event | sHR (95%CI) * | P value |
| Model 1 |  |  |  |  |  |  |  |  |  |
| Per SD increment | 225 | 1.19(1.07-1.32) | 0.001 | 51 | 1.15(0.89-1.48) | 0.299 | 59 | 1.23(1.02-1.49) | 0.032 |
| tertile 1 | 68 | 1.00(Ref.) | - | 14 | 1.00(Ref.) | - | 17 | 1.00(Ref.) | - |
| tertile 2 | 63 | 0.97(0.68-1.37) | 0.857 | 14 | 1.17(0.55-2.47) | 0.685 | 16 | 0.90(0.45-1.80) | 0.770 |
| tertile 3 | 94 | 1.64(1.18-2.27) | 0.003 | 23 | 2.46(1.23-4.93) | 0.011 | 26 | 1.61(0.85-3.04) | 0.147 |
| Model 2 |  |  |  |  |  |  |  |  |  |
| Per SD increment | 225 | 1.20(1.07-1.34) | 0.002 | 51 | 1.06(0.79-1.42) | 0.712 | 59 | 1.25(1.03-1.52) | 0.023 |
| tertile 1 | 68 | 1.00(Ref.) | - | 14 | 1.00(Ref.) | - | 17 | 1.00(Ref.) | - |
| tertile 2 | 63 | 0.96(0.68-1.36) | 0.812 | 14 | 1.02(0.48-2.16) | 0.966 | 16 | 1.00(0.50-2.01) | 0.998 |
| tertile 3 | 94 | 1.54(1.10-2.14) | 0.011 | 23 | 1.73(0.86-3.51) | 0.126 | 26 | 1.80(0.93-3.48) | 0.081 |
| Model 3 |  |  |  |  |  |  |  |  |  |
| Per SD increment | 225 | 1.25(1.08-1.45) | 0.003 | 51 | 1.18(0.83-1.68) | 0.361 | 59 | 1.38(1.08-1.78) | 0.011 |
| tertile 1 | 68 | 1.00(Ref.) | - | 14 | 1.00(Ref.) | - | 17 | 1.00(Ref.) | - |
| tertile 2 | 63 | 1.08(0.74-1.57) | 0.703 | 14 | 1.19(0.54-2.65) | 0.667 | 16 | 1.17(0.55-2.48) | 0.687 |
| tertile 3 | 94 | 1.70(1.13-2.56) | 0.010 | 23 | 2.48(1.05-5.88) | 0.039 | 26 | 2.28(1.02-5.08) | 0.045 |

*: sub-distribution hazard ratio accounting for the competing event

Model 1: unadjusted; model 2: adjusted for age, sex, ethnicity, smoking status, drinking status, and education; and model 3: further adjusted for BMI, SBP, DBP, albumin, total cholesterol, serum creatinine, serum potassium, Hemoglobin, MCV, diabetes, hypertension, CVD, cancer, and ferritin

**

**

**Supplemental Figure S1. The distribution of serum ferritin among study population**
